# Agonistic interactions and vocalizations among Procellariiformes in multi-species feeding aggregations, observed through bird-borne cameras

**DOI:** 10.1098/rstb.2025.0201

**Published:** 2026-10-15

**Authors:** Andréa Thiebault, Isabelle Charrier, Maëlle Amiot, Peter G Ryan, Pierre Pistorius, Stefan Schoombie

**Affiliations:** ^1^ Institut des Neurosciences Paris-Saclay (NeuroPSI), CNRS UMR 9197, Université Paris-Saclay, 91400 Saclay, France; ^2^ Marine Apex Predator Research Unit, Institute for Coastal and Marine Research, Nelson Mandela University, Gqeberha 6001, South Africa; ^3^ FitzPatrick Institute of African Ornithology, University of Cape Town, Rondebosch 7701, South Africa; ^4^ Department of Statistical Sciences, Centre for Statistics in Ecology, the Environment and Conservation (SEEC), University of Cape Town, Cape Town, South Africa; ^5^ La Rochelle Université, Laboratoire L3i, Faculté des Sciences et Technologies, Bâtiment Pascal Avenue Michel Crépeau, 17042, La Rochelle, France; ^6^ Centre D′Études Biologiques de Chizé, UMR7372 CNRS-La Rochelle Université, Villiers en Bois, France

**Keywords:** albatross, bioacoustics, biologging, ethology, competition, petrel, prion, visual display, resource sharing, Prince Edward Islands

## Abstract

Marine predators frequently aggregate at food sources, where interactions can range from cooperative group hunting to interference competition for resources. To investigate interactions at feeding aggregations among procellariiforms, we equipped wandering albatrosses *Diomedea exulans* with video cameras, collecting 43 hours of footage from 22 individuals. Cameras from eight birds recorded feeding aggregations, capturing a total of 62 minutes of footage (2.7% of observations). All events involved multiple species, including other wandering albatrosses, sooty albatrosses *Phoebetria* spp., giant petrels *Macronectes* spp., white-chinned petrels *Procellaria aequinoctialis* and smaller petrels (prions *Pachyptila* spp., blue petrels *Halobaena caerulea*, gadfly petrels *Pterodroma* spp. and storm petrels Oceanitidae). As in terrestrial bird assemblages, access to food was largely regulated by the size of competing individuals. The presence of other wandering albatrosses influenced interactions, with agonistic interactions in multi-species associations (intimidation postures, strikes and fights) observed only in events involving more than one wandering albatross (four of eight events). These interactions were accompanied by vocalizations. We identified five types of vocalizations from four individuals based on acoustic structure (*n* = 84 vocalizations). Intimidation postures were frequently accompanied by modulated vocalizations, indicating a combined visual-acoustic threat display. High-pitched screams, though rare, were also associated with agonistic interactions. These results contribute to a better understanding of foraging ecology and resource access in scavenging seabirds.

This article is part of the theme issue ‘Multi-predator feeding aggregations in pelagic waters’.

## 1. Introduction

The aggregation of predators often reflects the distribution of their prey [1,2] and can also be part of a social predation strategy [3]. When facing the challenge of having to share food sources, interactions among species and within species can integrate both positive (e.g. facilitation) and negative (e.g. competition) components [4–10]. For species living in stable groups, resource allocation is regulated by established dominance hierarchies: individuals gain access to food in order of rank mediated by social status, sex, breeding status and/or age (e.g. baboons [11], hyaenas [12], lions [13], bears [14] and crows [15,16]). When food attracts predators from different groups or species, competitive interactions often determine the division of resources [17,18]. Agonistic interactions include threat displays (e.g. intimidating postures), charges or attacks, fights or robbing of food [18,19]. In multi-species aggregations where predators regularly interact with the same species assemblages, dominance patterns may also arise with some species displaying more aggressive behaviours than others (e.g. crows [15], hawks [20]) or with larger individuals dominating smaller ones (e.g. various bird assemblages [18,21]). However, smaller species may gain advantage when hunting in groups, confronting competing species and defending access to food when in sufficient numbers (e.g. wild dogs [22,23] and coyotes [24]). Other parameters, such as the type or size of prey, also modulate the interactions among predators [17,25,26]. While such interactions and associated mechanisms have been abundantly studied in terrestrial systems, much less is known for animals foraging at sea.

The pelagic marine environment provides a variety of food sources, which are often patchily distributed [27]. Marine predators aggregate over food patches [28–30], a distribution pattern further amplified by social mechanisms, such as local enhancement [31–36]. When gathered at a patch, marine predators may feed with no apparent coordination (e.g. penguins feeding on krill [37,38]), or they may use group-hunting strategies to overcome the antipredator mechanism of schooling behaviour [10,39,40] (e.g. cetaceans [41–43], seabirds [44,45], sailfish [46] and pinnipeds [47]). In multi-species aggregations feeding on schooling prey, pursuit-diving species (typically marine mammals or penguins) feed from below the patch and push prey closer to the surface where they become more accessible to near-surface feeding species [33,48–50]. For other types of prey, when prey are scarce or when no specific hunting strategy is required to capture prey, individual competition can be expected to dominate interactions among predators for resource monopolization. This is typically the case for isolated prey, such as solitary fish or squid, or for carrion. For example, interference competition involving a range of agonistic interactions commonly occurs in multi-species assemblages of seabirds feeding on fisheries discards [51–53], and attempted food robbing has been reported in penguins feeding on squid [54]. As is typically found in terrestrial systems, dominance patterns tend to be related to body size [53,55], but some species may exhibit more aggressive behaviour independent of their size [52].

Many procellariiform seabirds have developed exceptional flying capacities [56–58], using the wind and waves for dynamic soaring to travel long distances at low-cost [56], thus exploring thousands of square kilometres in search of prey [59]. They feed near the sea surface (surface seizing, shallow diving and scavenging) on fishes, squid or crustaceans, depending on the species [60]. More specifically, the diet of wandering albatrosses primarily consists of fishes, cephalopods and crustaceans, with relative proportions varying by geographical location and individual sex [61–64]. They are considered scavengers [61,62,64] as they typically acquire floating and decaying prey at or near the sea surface. Occasionally, the diet of wandering albatrosses may also include carrion from cetaceans or other seabird species [55,61,62,64]. The large olfactory bulbs of procellariform seabirds give them remarkable abilities [65,66], which they use to navigate [67] and to locate productive areas over long distances [68]. Other seascape features, such as oceanographic fronts [69] or infrasound cues [70], may further be used for large-scale navigation. At a finer scale, their search for prey includes a combination of olfactory and visual cues [71], as well as local enhancement [72,73]. While at least some species benefit from social interactions to locate prey [74], the nature of their prey and feeding technique suggest they would not require a group strategy to capture prey, with competition being expected at food patches. Competition for resources is limited by mechanisms of spatial segregation that restrict overlap of foraging areas within species, depending on sex [75,76], age [77] or breeding location [78]. Nonetheless, overlap of foraging areas still occurs, raising questions of how Procellariiformes share and divide food sources. Observations at sea have indicated that large albatross species (*Diomedea* spp.) often aggregate with conspecifics when feeding, competing with giant petrels *Macronectes* spp. and dominating sooty albatrosses *Phoebetria* spp., highlighting size-dominating competition in these assemblages [55,79]. Interactions among feeding seabirds may, however, also involve vocal communication [80,81]. In procellariiforms, competitive interactions are accompanied by a range of vocalizations [79,82], calling for an acoustic description of these vocalizations.

In this study, we equipped wandering albatrosses *Diomedea exulans* breeding on Marion Island (Prince Edward Islands, southwestern Indian Ocean, 37° E, 46° S) with video cameras to observe feeding aggregations directly from the birds’ perspective. We describe the composition of aggregations (species present and numbers), the behaviour of individuals at the aggregation, the acoustic structure of vocalizations produced by wandering albatrosses and the interactions among and within species during these feeding events.

## 2. Material and methods

### (a) Data collection

Data were collected as part of a study looking at the fine-scale flight behaviour of wandering albatrosses [83]. Custom-made video cameras (77 × 34 × 18 mm; 48 g) were deployed on 33 adult wandering albatrosses that were incubating eggs or rearing small chicks between 2016 and 2020. Loggers were attached using waterproof Tesa tape (Beiersdorf) on the central dorsal contour feathers in line with the body axis of the birds. The video cameras recorded high-definition video (1280 × 720 pixels resolution) at 24 frames per second and were programmed to record 5–10 minutes of video at 3–6 hour intervals, or when the logger was immersed in salt water, with the exception of three deployments in 2020, which recorded video continuously until the battery was depleted. Some of the loggers had a light sensor, limiting filming to periods when there was enough light for visual observation. Additional loggers were deployed with the video cameras, but not used in the present study. The combined maximum mass attached to each bird (including waterproofing and attachment tape) was approximately 130 g, which is much less than 3% of the mass of typical adult wandering albatrosses (approx. 7000 g).

### (b) Video analyses

Video footage was analysed using BORIS software [84] to identify feeding events, species present and annotate behavioural interactions, adjusting the viewing speed (often frame by frame) as necessary. The recording duration of a feeding aggregation was calculated as the cumulative time the equipped bird spent at the centre of the aggregation, feeding and interacting with others (i.e. removing moments when the equipped bird was flying). As a consequence, the recording duration did not necessarily reflect the duration of the feeding events. Prey was identified when visible.

#### (i) Description of aggregations

Species present at an aggregation were identified to the lowest taxonomic level possible. Feeding individuals (other than the equipped bird) were annotated when clearly visible in the field of view. As such, species were considered in the centre of the aggregation if very close to the prey and seen feeding. Species were considered at the edge of the aggregation when individuals were on the water further away from the centre or flying around and over the prey. Birds flying in the distance were annotated, but not considered as part of the aggregation. Estimation of the number of individuals for each species present in an aggregation was based on the recording of the entire event. To avoid counting the same individual multiple times, the counting was done with one of the following methods: the highest number of individuals visible in a single frame, the cumulative count of individuals over a few consecutive frames, a global view of the aggregation recorded from the equipped bird when flying over the aggregation.

#### (ii) Behaviours and behavioural interactions

We annotated every moment another bird (apart from the equipped bird) was observed feeding, as well as every time a bird was flying over the prey. Three types of agonistic interactions were annotated: (i) strikes, which consisted of an individual launching its head and beak towards another individual, with species involved annotated; (ii) fights, which consisted of two individuals striking at each other and grabbing each other's beaks, with species involved; and (iii) intimidation postures, which consisted of the bird rising up (looking as if it was rearing), often associated with a call. Intimidation postures were only recorded for equipped albatrosses, as it was difficult to detect in other birds owing to their fleeting nature. The duration of fights and intimidation postures was recorded.

### (c) Acoustic analyses

This analysis was focused on data from six individuals for which we observed feeding events and that had associated sound recordings. Sound files (.wav format) were extracted from videos using GoldWave software (Newfoundland, Canada). Vocalizations were labelled and analysed using A visoft-SASLab Pro software (version 5.3.2-61; Avisoft Bioacoustics, Germany). The oscillogram and spectrogram (Hamming function, fast Fourier transform 1024 points window size, 87.5% overlap) of recordings were visualized over sliding windows to identify and label vocalizations. Vocalizations from wandering albatrosses were aurally and visually identified, using two recordings of wandering albatrosses feeding near a boat as a reference for species identification (xeno-canto.org recording XC796375 by Barry Edmonston and XC883164 by Greg McLachlan). Vocalizations were categorized according to their main acoustic characteristics (e.g. calls with harmonic structure, calls with frequency modulation and low or high pitch). Vocalizations from other species were annotated as ‘other’. Acoustic measurements were performed on vocalizations from wandering albatrosses, in order to quantify their acoustic structure. Only two acoustic variables were measured because the sound data were recorded from built-in microphones in the video cameras, embedded in a waterproof casing (thus modifying the energy distribution among frequencies and preventing us from taking additional measurements). The two acoustic variables measured were: (i) call duration (s) measured from the oscillogram, and (ii) fundamental frequency (Hz) measured manually for each call on the averaged amplitude spectrum, using the harmonic cursor tool on A visoft-SASLab Pro.

### (d) Behavioural associations

We quantified the temporal co-occurrence between video observations (intimidation posture, strike, fight, other bird feeding and other bird flying over the prey) and vocalizations by analysing the overlap of their time intervals. Analyses were conducted in two ways: assessing behaviours recorded during vocalizations, and the vocalizations recorded during social interactions. For the latter, vocalizations were counted as the cumulative number of recorded vocalizations (e.g. cumulating counts if several vocalizations were recorded in one event). For observations (behaviour or vocalization) that were defined with a start time and an end time, overlaps were systematically identified by comparing time intervals of each observation. For strikes (with no duration), vocalizations were counted from 0.5 s before to 0.5 s after the event. For fights (with a duration, but under-estimated because fights often went out of frame), vocalizations were counted from 0.2 s before to 0.2 s after the event.

## 3. Results

Of 33 wandering albatrosses equipped with video cameras, we obtained approximately 43 hours of useful footage from 22 birds, including 10 females and 12 males (average 116 ± 113 minutes per bird, range 10–425 minutes). At sea, wandering albatrosses spent most of their time flying or sitting on the water alone, regularly crossing the paths of other seabirds but not interacting with them. A feeding aggregation, as observed from the camera onboard wandering albatrosses, consisted of feeding behaviour in the equipped bird as well as in other surrounding birds. In these aggregations, agonistic interactions were intermixed with vocalizations. Feeding aggregations were recorded from eight individuals (one feeding event per individual), resulting in a total of 62 minutes of foraging footage.

All eight events involved a localized food source (seemingly single prey items). Wandering albatrosses fed on a variety of floating prey, including squid, fishes, another seabird's carrion and unidentified carrion (figure 1). All feeding events involved multi-species aggregations, including other species of albatrosses (sooty albatrosses *Phoebetria fusca*/*palpebrata*, in four of eight events), giant petrels *Macronectes giganteus*/*halli* (one bird in the centre of one aggregation), white-chinned petrels *Procellaria aequinoctialis* (six of eight events) and ‘small petrels’ (six of eight events). Small petrels included prions *Pachyptila* spp., blue petrels *Halobaena caerulea*, gadfly petrels *Pterodroma* spp. (mainly soft-plumaged petrels *Pterodroma mollis*) and storm petrels (mainly black-bellied storm petrels *Fregetta tropica*). Small petrels were grouped in a single category as they were more difficult to identify, often fleeting and farther away from the camera.

**Figure 1.**
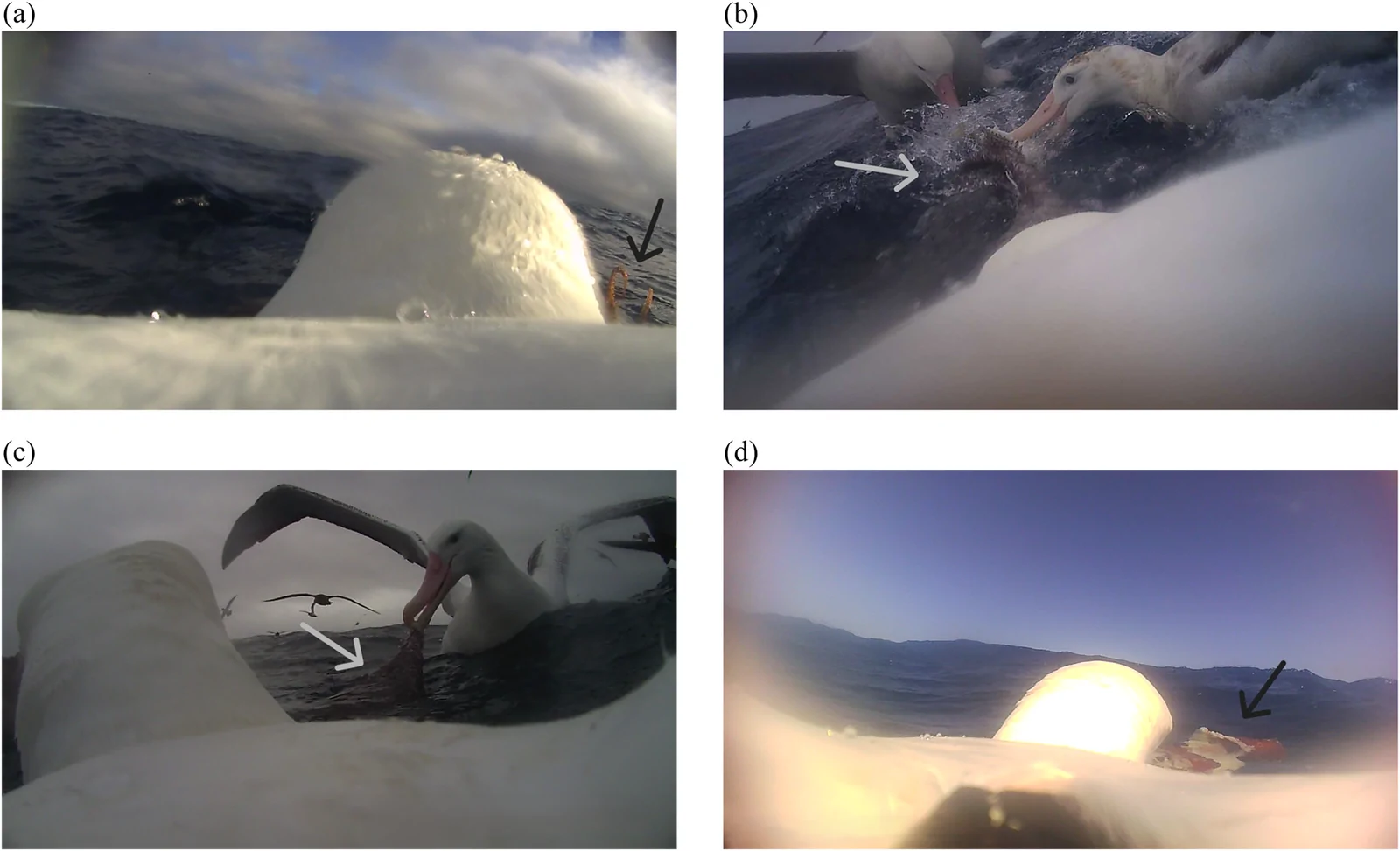
Frames extracted from video cameras where prey was visible: (a) squid from WA_2017_03, (b) fish from WA_2018_07, (c) bird from WA_2018_09, and (d) floating carcass from WA_2020_36.

The presence or absence of other wandering albatrosses influenced the interactions between individuals, with agonistic interactions only observed when more than one wandering albatross was present at the feeding aggregation (table 1). We thus distinguished between aggregations including a single wandering albatross (the focal bird) and aggregations including several wandering albatrosses.

**Table 1.** Description of feeding aggregations observed from a video camera attached on the back of wandering albatrosses. (Recording duration was calculated as the cumulative time the equipped bird spent at the aggregation. Species were identified at the lowest taxonomic level possible: WA, wandering albatross; SA, sooty albatross; WCP, white-chinned petrel; GP, giant petrel; SP, small petrels. Strikes and fights involve two wandering albatrosses, except when indicated. Vocalizations: mod, modulated; harm, harmonic; lowmod, low-pitched modulated; and modscream, modulated-scream. M, male. F, female. NV, not visible. NA, not available.)

type of aggregation	bird ID	sex	recording duration (s)	prey	aggregation		flying around the area	agonistic interactions			*n* vocalizations
					centre	edge		*n* strikes	*n* fights	*n* intimidation	
1 WA	WA_2020_35	F	56	NV	1 WA	1 SP		0	0	0	0
	WA_2017_05	M	300	NV	1 WA, 1 SA	3 WCP		0	0	0	0
	WA_2017_03	F	377	squid	1 WA, 4SA	2 WCP		0	0	0	nine mod; one lowmod; two harm; eight other
	WA_2020_36	M	958	carrion	1 WA	4 SP, 2 WCP		0	0	0	NA
multiple WA	WA_2017_08	M	80	NV	7 WA	5 WCP, 1 SP	6	1 WA-WA	2	0	16 mod; three modscream; 13 harm; three other
	WA_2018_07	M	459	fish carrion	8 WA	5 WCP, 20 SP		12WA-WA	4	11	NA
	WA_2018_09	M	737	NV bird carrion	5 WA, 4 SA, 12 WCP, 1 GP	10 SP	28	5 WA-WA, 2 WCP-WCP	1	23	65 mod; one modscream; three scream; eight lowmod; 14 harm; 29 other
	WA_2016_04	M	739	NV	6 WA	1 SA, 9 SP		16 WA-WA	5	43	283 mod; 24 modscream; six scream; 135 lowmod; 221 harm

### (a) Aggregations with only one wandering albatross

Four events involved the focal wandering albatross (two females and two males) feeding in association with other species. All, except one very short event, included white-chinned petrels flying around and over the prey, sometimes landing nearby and feeding farther away, seemingly on scraps (figure 2a). Two events involved sooty albatrosses, sitting on water nearby, facing and looking towards the equipped bird feeding, also feeding on scraps (figure 2b). The two other events included small petrels (gadfly petrels and prions) flying around the area, sometimes flying over the prey or briefly surface-feeding nearby (figure 2c). No agonistic interactions were observed in these cases. In one event, a few vocalizations were recorded both from the equipped bird (approx. 3 vocalizations min^−1^) and from surrounding birds (possibly from sooty albatrosses).

**Figure 2.**
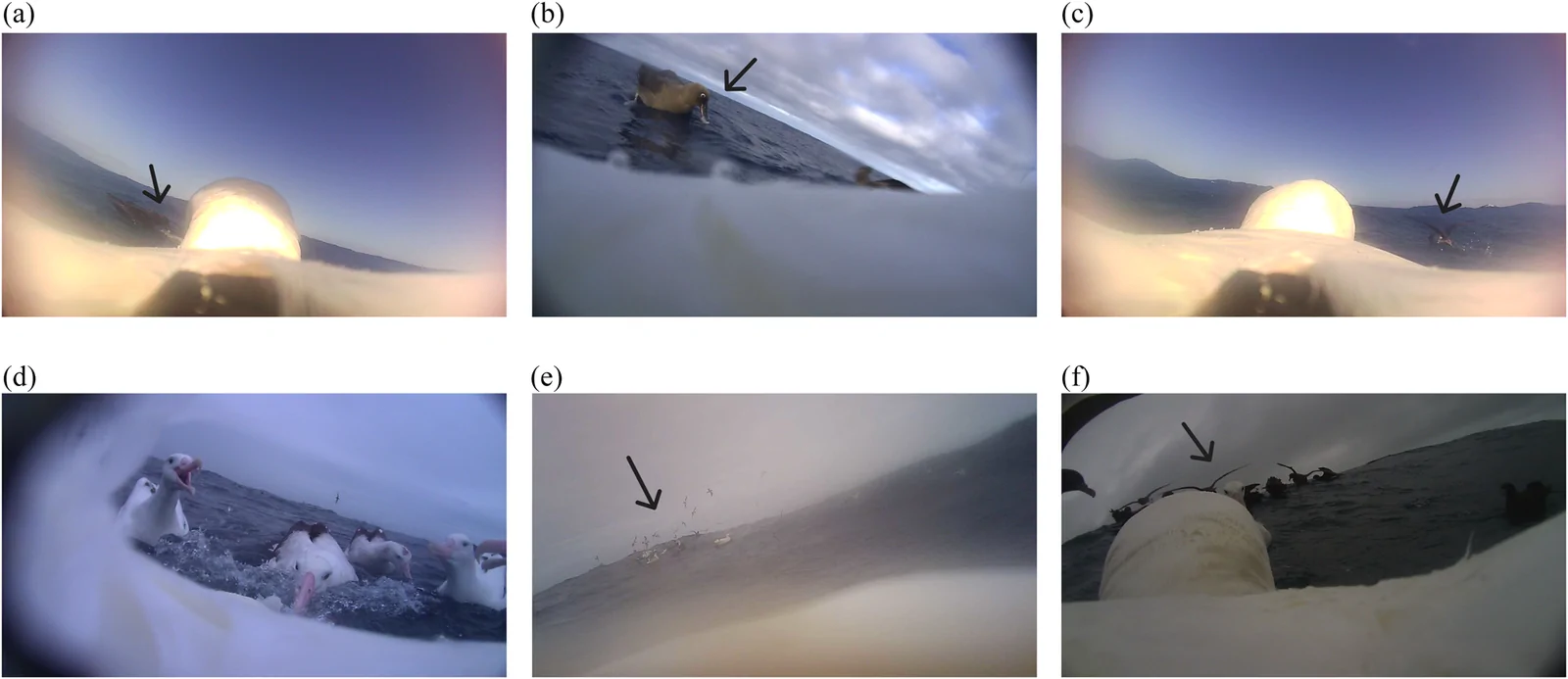
Frames extracted from video cameras onboard wandering albatrosses, showing multi-species feeding aggregations. The top row shows events, involving a single wandering albatross, where other species are seen feeding: (a) white-chinned petrel from WA_2020_36, (b) sooty albatross from WA_2017_03, and (c) small petrels from WA_2020_36. The bottom row shows events involving several wandering albatrosses: (d) six albatrosses aggregated over prey, from WA_2017_08, (e) multi-species feeding aggregation observed from a distance, from an albatross in flight, from WA_2018_07, and (f) 11 white-chinned petrels aggregated over prey, another wandering albatross also visible in front, from WA_2018_09.

### (b) Aggregations with several wandering albatrosses

We recorded four events where five to eight wandering albatrosses competed for the same prey (figures 1b and 2d,e). These events were all recorded from males (equipped birds) and involved birds in a range of plumages (Gibson stages 4–7, following [85]), but no juveniles. Two events included sooty albatrosses sitting on the water nearby, facing the aggregation and sometimes feeding, and in one instance a giant petrel was feeding in the centre of the aggregation. White-chinned petrels were observed in three events, in larger numbers compared with events with a single wandering albatross, reaching greater than 10 individuals in one event (table 1; figure 2f). Up to 20 small petrels (including prions, blue petrels, soft-plumaged petrels and storm petrels) were recorded close to the feeding group, and in two events big flocks of petrels were observed farther away. Several agonistic interactions were recorded in these events, at a rate of 1–3 interactions min^−1^. These involved mostly wandering albatrosses but a few also involved white-chinned petrels (table 1). Vocalizations were much more frequent than in single-albatross events, with 10–54 vocalizations min^−1^.

### (c) Description of vocalizations

Of the six individuals included in these analyses, we recorded 84 vocalizations during feeding events from four equipped individuals. We identified five types of wandering albatross vocalizations (figure 3), lasting 0.06–4.18 s and with fundamental frequencies of 40–544 Hz (table 2). The two most common were harmonic vocalizations (short duration and displaying a clear harmonic structure) and modulated vocalizations (longer duration and combined amplitude-frequency modulations). We also recorded low-pitched modulated vocalization, characterized by a low fundamental frequency. Sometimes, modulated vocalizations were associated with a ‘scream’ part, and more rarely scream vocalizations were recorded on their own. Scream vocalizations were characterized by an increase in frequency, the fundamental frequency doubling on average compared with modulated vocalizations (table 2).

**Figure 3.**
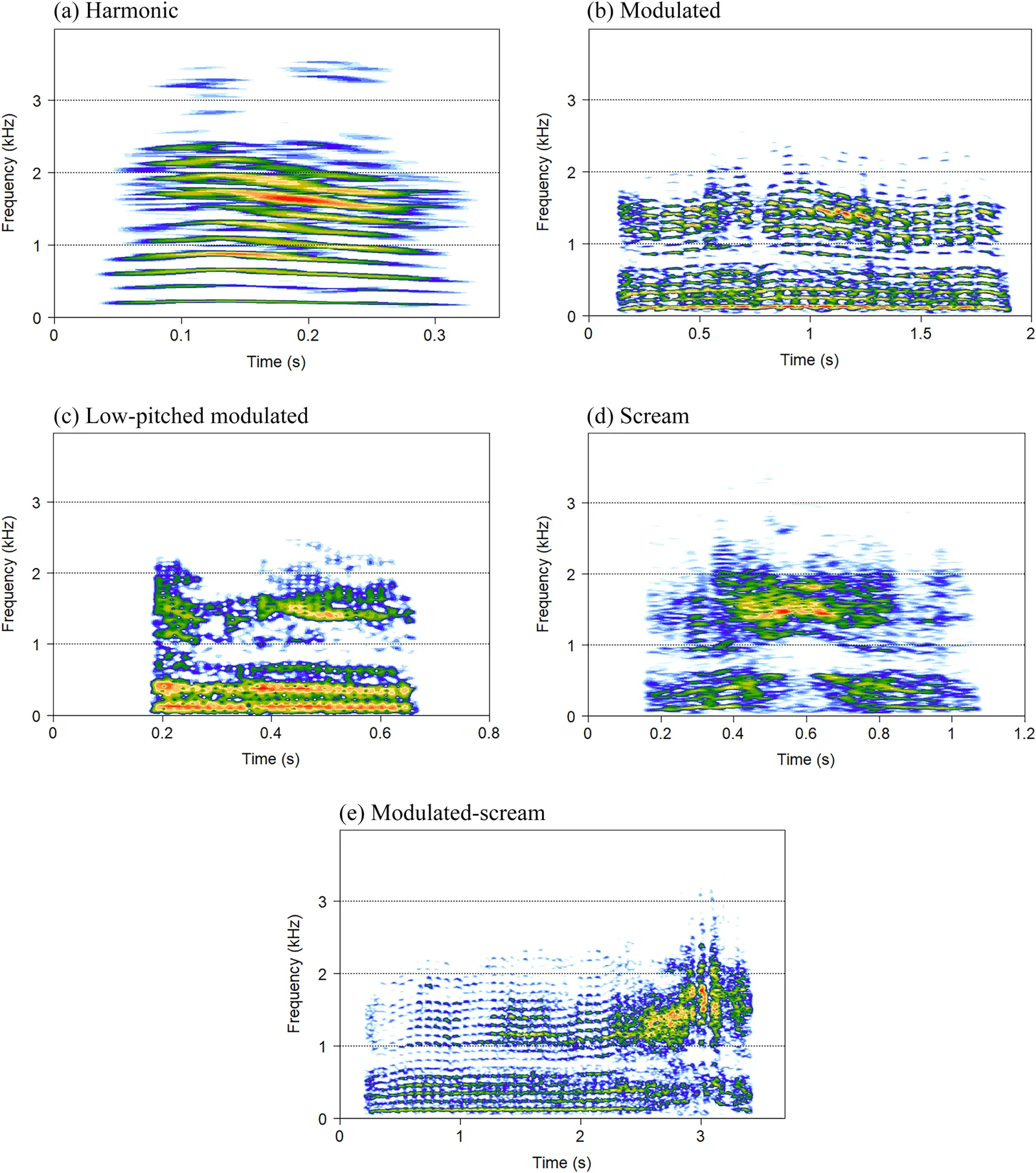
Spectrograms (frequency over time) of the five types of wandering albatross vocalizations recorded by onboard video cameras: (a) harmonic, (b) modulated, (c) low-pitched modulated, (d) scream, and (e) modulated-scream. Audio recordings (wav files) are provided in the supplementary material, audio.

**Table 2.** Acoustic measurements on five types of vocalizations recorded from video cameras onboard wandering albatrosses. (Data were recorded during feeding events from four different individuals (*n* = 844).)

vocalization type	duration (s)			fundamental frequency (Hz)			associated behaviours	
	*n*	mean ± s.d.	range	*n*	mean ± s.d.	[min; max]	*n* calls	*n* behaviours
harmonic	250	0.45 ± 0.26	[0.12–1.72]	236	144 ± 39	[80; 363]	22	six fly over; eight other feeding; five intimidation; six strikes
modulated	373	1.13 ± 0.57	[0.15–3.71]	313	119 ± 18	[64; 194]	77	23 fly over; 22 other feeding; 36 intimidation; six strikes; four fights
low-pitched modulated	144	0.38 ± 0.30	[0.06–2.42]	115	70 ± 22	[40; 136]	9	three fly over; five other feeding; one intimidation
modulated-scream	28	1.80 ± 0.92	[0.73–4.18]	26	113 ± 10	[95; 132]	9	two fly over; two other feeding; five intimidation; one strike; one fight
scream	9	0.77 ± 0.29	[0.38–1.24]	4	229 ± 210	[111; 544]	5	one intimidation; three strikes; one fight

Modulated vocalizations were mostly associated with intimidation postures, other birds flying over the prey and/or other birds seen feeding, sometimes with a combination of a potential threat from other birds (bird flying over or seen feeding) and the equipped bird taking an intimidation posture (table 2). Scream vocalizations (either alone or associated with a modulated vocalization) were only recorded in events with several wandering albatrosses aggregated (table 1). Among the vocalizations for which we could observe an associated behaviour, the majority of scream vocalizations were recorded during agonistic behaviours (six out of 14 intimidation postures, four out of 14 strikes or two out of 14 fights).

### (d) Social interactions

Agonistic interactions among albatrosses included intimidation postures, attacks and fights. As such, a feeding event was quantified as a succession of behaviours, including feeding, threats and agonistic interactions, intermixed with vocalizations (figure 4).

**Figure 4.**
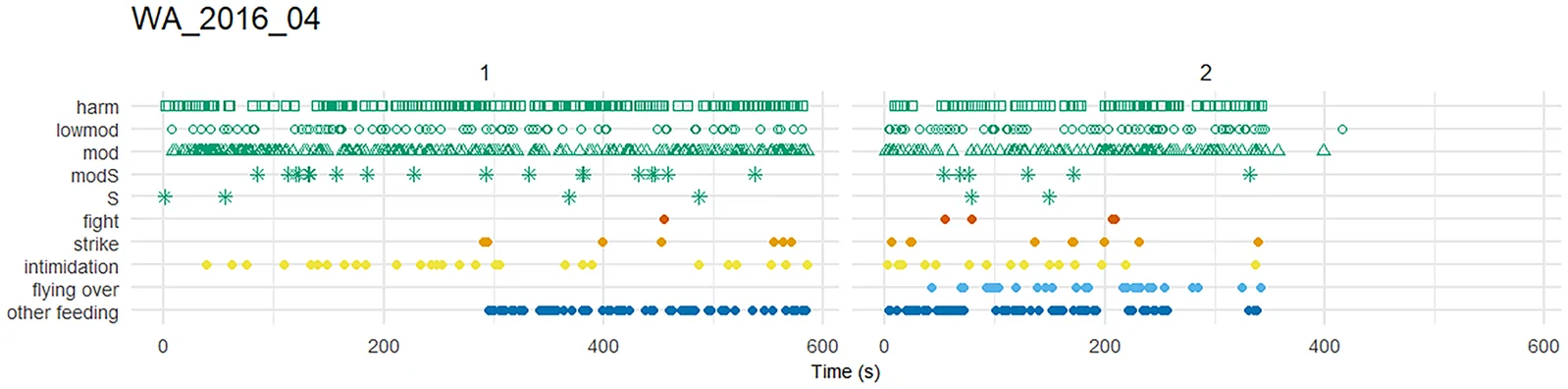
Behavioural sequence observed from onboard video cameras over time. Vocalizations in green: harmonic (harm), low-pitched modulated (lowmod), modulated (mod), modulated-scream (modS), scream (S). Agonistic interactions: fights in red, strikes in orange and intimidation postures in yellow. Birds flying over the prey in light blue (typically white-chinned petrels, small Procellariidae and Oceanitidae). Individuals other than the equipped bird feeding (dark blue).

Three birds made intimidation postures (supplementary material, video S1) in feeding aggregations involving other wandering albatrosses. The postures occurred at a rate of 1–3 min^−1^ and lasted 3.6 ± 2.3 s (table 3). Different types of behaviours from other birds were recorded in the second preceding the intimidation posture: birds flying over, other birds feeding and strikes (table 3). Four intimidation postures were followed by a strike and three by a fight. Most intimidation postures were associated with vocalizations (85%, *n* = 56 of 66 with sound data). Most were modulated vocalizations, followed by harmonic vocalizations (table 3). Scream vocalizations (alone or modulated-scream) were recorded during seven intimidation postures.

**Table 3.** Agonistic behavioural interactions recorded from video cameras onboard wandering albatrosses during multi-species feeding aggregations. (*n* for vocalizations differ from *n* total because two recordings lack sound data. Durations are expressed as mean ± s.d. [range], but are minimum estimates because fights often went out of frame.)

	*n*	duration (s)	behaviour during 1 s before	behaviour during 1 s after	*n* (data with sound)	associated vocalizations
intimidation	77	3.6 ± 2.3 [0.7–10.4]	seven fly over; seven other feeding; four strikes	six fly over; 12 other feeding; four strikes; three fights	66	42 harm in 26 events; 94 mod in 54 events; five modscream in four events; three scream in three events; 15 lowmod in 12 events
strike	36	NA	six fly over; seven other feeding; one intimidation	seven fly over; three other feeding; five intimidation	24	11 harm in nine events; six mod in six events; one modscream in one event; two scream in two events
fight	12	1.4 ± 0.9 [0.2–2.9]	two fly over; two other feeding	one fly over; three intimidation	8	one harm in one event; four mod in three events; one modscream in one event; one scream in one event

Strikes were observed in all four feeding aggregations involving several wandering albatrosses, at a rate of 1–2 strikes min^−1^. Most were made between wandering albatrosses (*n* = 34, figure 5a), but two were made between white-chinned petrels (figure 5b). Again, different types of behaviours were recorded from other birds in the second preceding a strike: birds flying over, other birds feeding and intimidation postures (table 3). Five strikes were followed by an intimidation posture (supplementary material, video S1). Vocalizations were recorded during 67% of strikes (16 of 24 strikes for which sound was recorded). These included mostly harmonic vocalizations, although scream vocalizations were recorded in association with three strikes (table 3).

**Figure 5.**
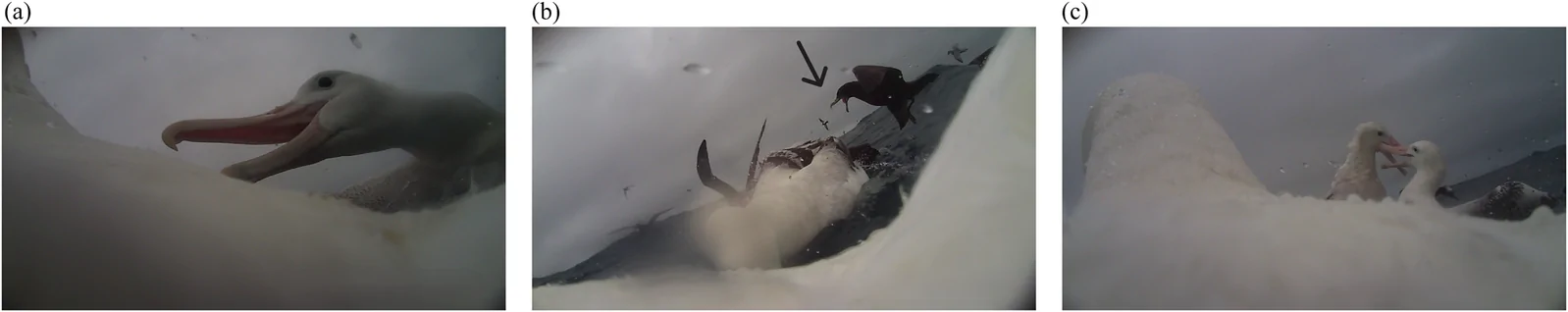
Strikes as observed from video cameras onboard wandering albatrosses: (a) from another wandering albatross towards the equipped bird from WA_2018_09, and (b) between two white-chinned petrels from WA_2018_09. (c) Fight between two albatrosses in front of the equipped bird from WA_2018_09.

Although rarer than other agonistic interactions (*n* = 12), fights between wandering albatrosses were observed in all four events where several albatrosses competed for food. Five fights involved the equipped bird, with three immediately followed by an intimidation posture from the equipped bird. Fights were often associated with vocalizations (six out of eight fights with sound data), including two scream vocalizations (table 3).

## 4. Discussion

We analysed the behaviour and social interactions of foraging wandering albatrosses from onboard video cameras. Our observations were restricted to daylight hours (video cameras with light sensors), so they only report on daylight behaviours and missed the night component of the albatross feeding strategy [79,86,87]. The albatrosses spent the largest part of their time flying and preening solitarily and feeding represented only 2.7% of observations. However, when feeding, we always observed the equipped birds in multi-species aggregations, including other species of Procellariiformes. Our observations corroborate the image of solitary wandering albatrosses soaring and travelling thousands of kilometres at sea, but this image does not hold for when they are feeding. Procellariiformes follow a variety of cues to locate food, detecting prey odours over long distances [68] and visually cueing on foraging birds [72,74], leading to them aggregating over prey.

The diet of wandering albatrosses primarily consists of fishes, cephalopods and crustaceans [61–64], and occasionally may also include carrion from cetaceans or other seabird species [55,61,62,64]. Our observations, encompassing cephalopods, fishes, birds and unidentified carrion, align with the diversity of prey items previously documented for this species. In all cases, we observed albatrosses feeding at the sea surface on floating prey. As a consequence, the behavioural interactions that we described here appear independent of prey species and seem characteristic of near-surface-feeding Procellariiform aggregations. However, prey size may influence these interactions, as larger prey items could attract predators in larger numbers owing to local enhancement [72].

Several species of procellariiform seabirds aggregated around prey. Although our observations were limited by the field of view of the camera, it was clear that individuals engaged in agonistic behaviours for access to food. Repeated agonistic interactions among the same individuals or species may lead to the establishment of dominance patterns [88]. Confirming previous observations at sea [55,79], and similarly to terrestrial bird assemblages [18,21], access to the resource was primarily related to the size of individuals. The wandering albatross was the largest species in the aggregations (7–9 kg) and was always present at the centre, getting primary access to food. Sooty albatrosses (approx. 2.5–3 kg) and giant petrels (approx. 3.5 kg) were observed sitting on water nearby, facing the centre of the aggregation, sometimes feeding a couple of metres away. White-chinned petrels (approx. 1.3 kg) were more mobile, sitting on water nearby or flying low over the centre of the aggregation, to attempt to grab a piece of food. We did not record white-chinned petrels plunging into the group of albatrosses as previously reported [79], but in one instance, a white-chinned petrel flew so low that it almost collided with a wandering albatross. The presence of flying white-chinned petrels occasionally triggered intimidation postures and threat vocalizations, confirming they also interacted directly with albatrosses. Furthermore, agonistic interactions were recorded between white-chinned petrels. The smallest species, grouped here as small petrels (all < 500 g), were mainly flying around the aggregation and tended to remain at more of a distance. Smaller species flying over food probably manage to access food through greater agility [58,79].

We recorded various types of agonistic behaviours (threats including intimidation postures and vocalizations, attacks/strikes and fights), mostly performed by wandering albatrosses but also by white-chinned petrels, as previously observed [79]. Intimidation postures by wandering albatrosses consisted of the birds rearing up, suggesting it could be a display of size and serve a function of threat for other individuals to remain away from the food source [19]. Threat displays were the most frequently recorded behaviours, showing they were efficient in regulating interactions among feeding individuals, who only rarely engaged in more intensive behaviours, such as attacks and fights. Agonistic interactions from wandering albatrosses seemed to be mostly directed towards conspecifics, since we recorded these behaviours exclusively when several albatrosses were aggregated at the food source (except for vocalizations). However, it appeared evident that these behaviours also influenced other species that remained further away from the centre of the aggregation. We recorded only male wandering albatrosses in aggregations with multiple albatrosses, where agonistic behaviours were common. Nonetheless, we recorded threat vocalizations from a female interacting with sooty albatrosses and white-chinned petrels, showing they may also engage in agonistic interactions when feeding. On land, fights mostly occur between same sex individuals, and females engage in fights with other females when displaying with the same male [89]. More observations will be necessary to confirm if male wandering albatrosses do engage more regularly in agonistic feeding aggregations than females.

Agonistic interactions among wandering albatrosses were associated with various types of vocalizations. This compares with their behaviour on land that is also largely accompanied by vocalizations [89,90]. Intimidation postures were often associated with modulated vocalizations, suggesting the threat display included a combination of visual and acoustic signals as commonly observed in birds [91]. Vocalizations were recorded in very high numbers (in higher numbers than other types of agonistic behaviours), suggesting that it could serve as a buffer before albatrosses engage in physical agonistic interactions. Vocalizations could inform on individuals’ intention to get access to food and provide information on the individuals’ fighting ability [92], since acoustic parameters are often directly correlated with the size of individuals [93,94]. Scream vocalizations were only recorded in aggregations with several albatrosses. Changes in acoustic structure, such as the increase in frequency in scream or modulated-scream vocalizations, could reflect changes in emotional arousal [95]. Indeed, vocalizations terminating with a scream were interpreted as a particularly excitable performance in wandering albatrosses’ courtship displays [89]. In the context of predators fighting for food, these could be associated with particularly intense fights. In addition to short-range functions, the constant production of vocalizations in feeding aggregations generates acoustic cues that may travel and inform other individuals in the area of the presence of food, as shown in marine and terrestrial systems [41,96]. Although additional studies will be necessary to determine the biological function of at-sea vocalizations, the fact that they were recorded almost continuously at aggregations suggests that vocalizations play an important role in regulating feeding interactions among albatrosses.

Overall, procellariiform seabirds are involved in social foraging in the sense that they regularly interact with others when foraging [97], including an interplay of positive and negative interactions. At the scale of food searching, these pelagic species navigate through very large spaces. Mechanisms of spatial segregation restrict overlaps of foraging areas within species [75,77,78], limiting interference competition for the resources. Nonetheless, foragers can benefit from interactions with other species through mechanisms of facilitation to locate prey [72,74], leading to feeding aggregations. At the scale of feeding, once prey is located, the daylight behavioural interactions among species are mostly agonistic, as shown in this study. In the framework of social predation proposed by [3], procellariiforms would fit in a low level of social predation, benefiting prey searching but interacting agonistically for resource sharing. The interplay between positive and negative mechanisms may lead to different species undertaking different roles in the community [9], and could theoretically explain the coexistence of various species exploiting dynamic resources [48,98]. This suggests a fragile equilibrium for species facing population and environmental changes that impact resource distribution [99,100].

## Supplementary Material



## Data Availability

Data (videos and annotations) are available at the following repository [101]. Supplementary material is available online [102].

## Theme

One contribution of 14 to a theme issue ‘Multi predator feeding aggregations in pelagic waters’.
